# From Birdsong to Human Speech Recognition: Bayesian Inference on a Hierarchy of Nonlinear Dynamical Systems

**DOI:** 10.1371/journal.pcbi.1003219

**Published:** 2013-09-12

**Authors:** Izzet B. Yildiz, Katharina von Kriegstein, Stefan J. Kiebel

**Affiliations:** 1 Max Planck Institute for Human Cognitive and Brain Sciences, Leipzig, Germany; 2 Group for Neural Theory, Institute of Cognitive Studies, École Normale Supérieure, Paris, France; 3 Humboldt University of Berlin, Department of Psychology, Berlin, Germany; 4 Biomagnetic Center, Hans Berger Clinic for Neurology, University Hospital Jena, Jena, Germany; Institut de Neurosciences des Systèmes, France

## Abstract

Our knowledge about the computational mechanisms underlying human learning and recognition of sound sequences, especially speech, is still very limited. One difficulty in deciphering the exact means by which humans recognize speech is that there are scarce experimental findings at a neuronal, microscopic level. Here, we show that our neuronal-computational understanding of speech learning and recognition may be vastly improved by looking at an animal model, i.e., the songbird, which faces the same challenge as humans: to learn and decode complex auditory input, in an online fashion. Motivated by striking similarities between the human and songbird neural recognition systems at the macroscopic level, we assumed that the human brain uses the same computational principles at a microscopic level and translated a birdsong model into a novel human sound learning and recognition model with an emphasis on speech. We show that the resulting Bayesian model with a hierarchy of nonlinear dynamical systems can learn speech samples such as words rapidly and recognize them robustly, even in adverse conditions. In addition, we show that recognition can be performed even when words are spoken by different speakers and with different accents—an everyday situation in which current state-of-the-art speech recognition models often fail. The model can also be used to qualitatively explain behavioral data on human speech learning and derive predictions for future experiments.

## Author Summary

Neuroscience still lacks a concrete explanation of how humans recognize speech. Even though neuroimaging techniques are helpful in determining the brain areas involved in speech recognition, there are rarely mechanistic explanations at a neuronal level. Here, we assume that songbirds and humans solve a very similar task: extracting information from sound wave modulations produced by a singing bird or a speaking human. Given strong evidence that both humans and songbirds, although genetically very distant, converged to a similar solution, we combined the vast amount of neurobiological findings for songbirds with nonlinear dynamical systems theory to develop a hierarchical, Bayesian model which explains fundamental functions in recognition of sound sequences. We found that the resulting model is good at learning and recognizing human speech. We suggest that this translated model can be used to qualitatively explain or predict experimental data, and the underlying mechanism can be used to construct improved automatic speech recognition algorithms.

## Introduction

Can we learn something about how humans recognize speech from how birds recognize song? The last common ancestor of humans and birds lived about 300 million years ago, nevertheless human and songbird communication share several striking features at the cognitive, neuronal and molecular level [1], [2]. When we recognize speech, our brains map fast speech sound wave modulations to spectrotemporal auditory representations [3], [4]. Similarly, songbirds map song sound wave modulations to specific internal representations [5], [6]. In addition, similar to humans, songbirds gain their vocal abilities early in life by listening to adults, and memorizing and practicing their songs [2]. The similarities include anatomical and functional features that characterize the pathways for vocal production, auditory processing and learning [1], [2], [7]. For example, the auditory system in both humans and songbirds is organized hierarchically [8]–[10] where fast time scales are represented by lower levels and slow time scales by levels higher up in the hierarchy [11], [12]. Much more is known experimentally about the exact neuronal mechanisms in songbirds than in humans, due to detailed electrophysiological studies which have shown that songbirds use a sequence of auditory dynamics to generate and recognize song in a highly effective manner [6], [13]. These detailed findings in songbirds enabled us to derive a neurobiologically plausible, computational model of how songbirds recognize the songs of their conspecifics [14]. Our aim in the present paper is to attempt to translate this birdsong model to human speech by assuming that humans and birds use similar internal models for recognizing sounds. Such a translation would provide a unique opportunity to derive a mechanistic understanding and make predictions at both the microscopic and macroscopic neuronal level for the human speech learning and recognition system.

**Figure 1:**
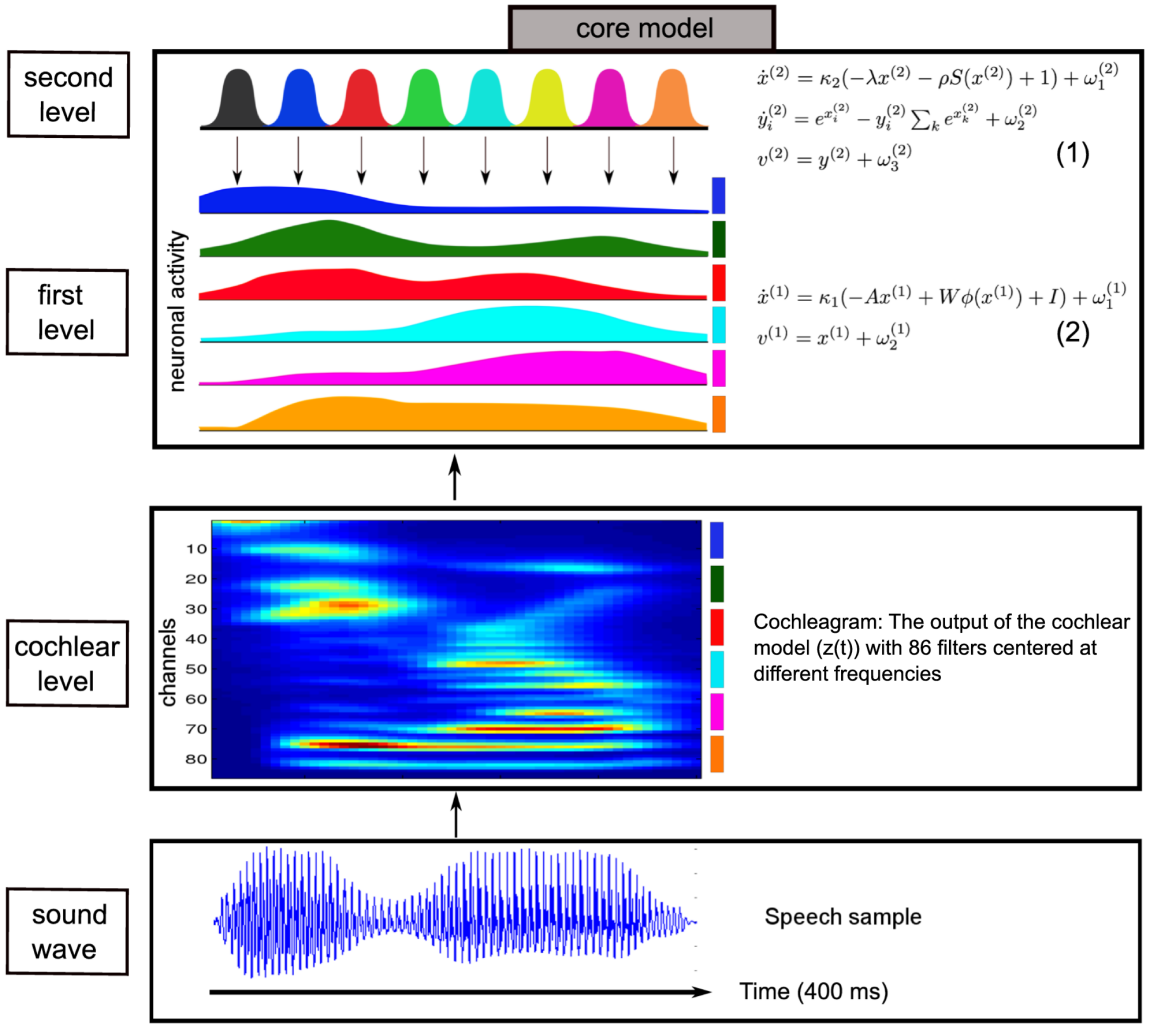
Summary of the hierarchical model of speech learning and recognition. The core of the model is equivalent to the core of the birdsong model [14]. The Equations 1 and 2 on the right side generate the dynamics shown on the left side, and are described in the Model section (see also Table 1 for the meaning of parameters). Speech sounds, i.e., sound waves, enter the model through the cochlear level. The output is a cochleagram (shown for the speech stimulus “zero”), which is a type of frequency-time diagram. There are 86 channels, which represent the firing rate (warm colors for high firing rate and cold colors for low firing rate) of the neuronal ensembles that encode lower frequencies as the channel number increases. We decrease the dimension of this input to six dimensions by averaging every 14 channels (see the color coding to the right of the cochleagram and also see Model). After this cochlear processing, activity is fed forward into the two-level hierarchical model. This input is encoded by the activity of the first level network (shown with the same color coding on the right), which is in turn encoded by activity at the second level (no color coding at this level, different colors represent different neuronal ensembles). From the generative model shown here (core model), we derived a recognition model (for mathematical details see Model).

The birdsong model described in [14] performs a Bayesian version of dynamical, predictive coding based on an internal generative model of how birdsong is produced [15]. The core of this generative model consists of a two-level hierarchy of nonlinear dynamical systems and is the proposed mechanistic basis of how songbirds extract online information from an ongoing song. We translated this birdsong model to human sound recognition by replacing songbird related parts with human-specific parts (Figure 1). This included processing the input with a human cochlea model, which maps sound waves to neuronal activity. The resulting model is able to learn and recognize any sequence of sounds such as speech or music. Here, we focus on the application of the model on speech learning and recognition. The contribution of this article is threefold: First, inspired by songbird circuitry, it proposes a mechanistic hypothesis about how humans recognize speech using nonlinear dynamical systems. Secondly, if the resulting speech recognition system shows good performance, even under adverse conditions, it may be used to optimize automatic speech recognition. Thirdly, the neurobiological plausibility of the model would allow it to be used to derive predictions for neurobiological experiments.

## Model

Here, we first describe the model conceptually, followed by mathematical details of the generative model, cochlear model, online Bayesian recognition and further details of the simulations described in Results.

### Conceptual overview: A generative model of human speech

As a model, we employ a novel Bayesian recognition method of dynamical sensory input such as birdsong and speech. The Bayesian approach first requires building of a so-called generative (internal) model, which is then converted to a learning and recognition model. The key advantage of this approach, as opposed to standard models in both human speech recognition and automatic speech recognition, is that the generative model is formulated as hierarchically structured, nonlinear dynamical systems. This means that one can employ generative models specifically tailored to birdsong or speech recognition. As we show in the following, this feature is crucial for translating experimental birdsong results to a concrete recognition model. This translation would not be possible with generic models such as are standard and widely used in automatic speech recognition, e.g. the hidden Markov model and, very recently, deep belief networks and liquid state machines [16]–[18]. Our model has also several differences from the influential models such as TRACE [19] and Shortlist [20], [21] and we provide a more detailed comparison in the Discussion.

In the birdsong model, we used experimental insights about the firing patterns of the premotor area HVC (formerly known as the high vocal center) and the nucleus RA (robust nucleus of the arcopallium) to derive a hierarchical song generation model [14]. In the high level structure HVC, specific neurons called HVC_(RA)_, fire sequentially at temporally precise moments [13], [22], [23] where each neuron of this sequence fires only once during the song to provide input to a group of RA neurons.

**Figure 2:**
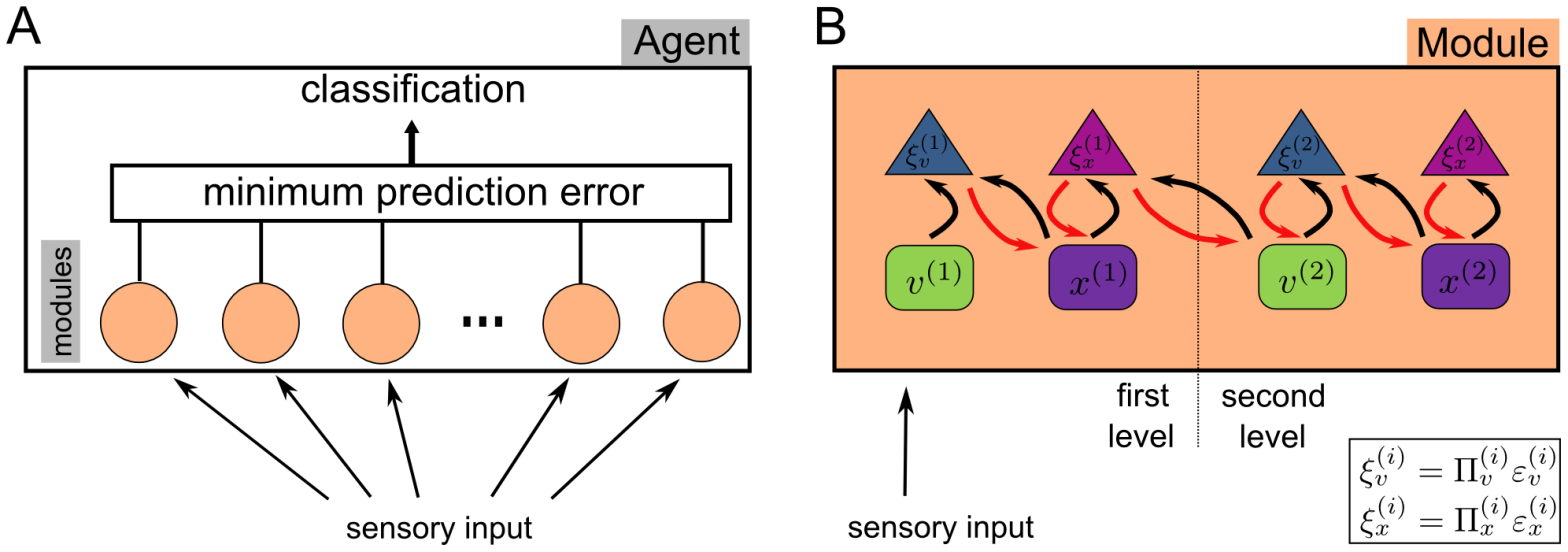
Schematic structure of an agent and a module. **A**) An agent consists of several modules, where each module contains an instance of the model shown in Figure 1 and has learned to recognize a single word. Sensory input is recognized by all modules concurrently and each module experiences prediction error during recognition. A module can be considered as a sophisticated dynamic, Bayes-optimal template matcher which produces less prediction error if the stimulus matches better to the module's learned word. A minimum operator performs classification by selecting the module with the least amount of prediction error during recognition. **B**) At each level in a module, causal and hidden states (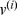 and 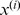, respectively) try to minimize the precision-weighted prediction errors (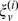 and 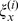) by exchanging messages. Predictions are transferred from second level to the first and prediction error is propagated back from the first to the second level (see section Model: Learning and Recognition for more details). Adapted from [110].

We translated these two levels to the human speech model in the present study (Figure 1). The second, higher level encodes a recurrent neural network producing a sequential activation of neurons in a winner-less competition setting (stable heteroclinic channels [24], see below). These dynamic sequences control dynamics at a first, lower level (Hopfield attractor, see below), where we model amplitude variations in specific frequency bands. In comparison to the birdsong model, the generative model here does not explicitly model the vocal tract dynamics but rather the dynamics at the cochlea which would be elicited by the stimulus. Therefore, the second level dynamics act as a timing mechanism providing the temporal information and the first level dynamics represent the spectral content at different frequency bands. Such a separation of temporal and spectral processing is also suggested for the human auditory system [25]. We do not restrict the functionality of the second level ensembles to specific phonemes or syllables but rather use them as time markers for the represented spectrotemporal stimulus (mostly words in this paper). By using this generative model (Figure 1), we can apply Bayesian inference to derive a mechanism, which can learn and recognize a single word. We call this mechanism for the remainder of this paper a *module*. Here, a module is essentially a sophisticated template matcher where the template is learned and stored in a hierarchically structured recurrent neural network and compared against a stimulus in an online fashion. Individual modules can be combined into an *agent* to achieve classification tasks as shown in the “Word Recognition Task” below, see Figure 2A for an overview. A crucial parameter in the model is called precision which is the inverse of the variance of an internal state. This is used in the model as a way to balance the (top-down) prior information and (bottom-up) sensory evidence. In the simulations, we show that the precision settings are crucial to learn new stimuli or to recognize sounds in noisy environments. We further discuss the biological plausibility of the resulting recognition model in the Discussion.

### Mathematical details: A generative model of human speech

#### Second level: Sequential dynamics

One of the well-established ways for modeling the sequential activation of neuronal ensembles is the Lotka-Volterra type dynamics [26], [27], which is well known in population biology. Rabinovich et al. applied this idea to neuronal dynamics under the name of winnerless competition [24], [27]–[30]. In the winner-less competition setting, there are 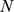 equilibrium points, i.e., neuronal ensembles, which are saddles of a nonlinear dynamical system. Each of these equilibrium points has a single unstable direction that connects them to the next equilibrium point while remaining directions are stable forming a so-called stable heteroclinic channel. In the phase space, this looks like beads on a string, which attracts nearby orbits. Therefore, a typical solution of such system with a heteroclinic contour travels through all saddle points, i.e., neuronal ensembles, in a circular fashion thereby activating each ensemble for a brief period until it is deactivated as the next ensemble becomes active.

These dynamics can be obtained from a neural mass model of mean membrane potential and action firing potential [31], reviewed in [24]. We use the following equations (see Table 1 for the constants used):
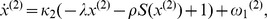


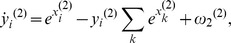


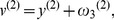
(1)where 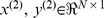 are the *hidden-state* vectors (e.g., mean membrane potentials) at the second level, 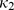 and 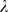 are scalars, 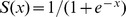 is the sigmoid function applied component-wise and 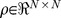 is the connectivity matrix with entries 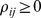 giving the strength of inhibition from state 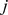 to 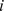. While the first set of hidden states, 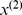, describes the heteroclinic channel, the second set of hidden states, 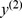, acts as smooth normalizing dynamics for 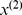 by limiting their dynamics to the interval 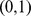. The states 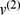 are called *causal states* and are used to transmit the output of the second level to the first level where this transformation is taken as identity here. We also add normally distributed noise vectors 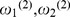 and 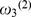 to render the model stochastic. Note that we use exponential functions in the dynamics of 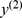 to decrease the overlaps between the dynamics of two sequentially activated neurons. A simpler normalization function such as the logistic function would give mostly overlapping activations which would be problematic during recognition. Therefore, each neuron can be considered to be highly sensitive to other neurons' firing rates since even a slight activation of one neuron quickly suppresses (due to exponential function) the activation of all other neurons in the network. With an appropriately chosen connectivity matrix, one can obtain a system with 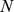 saddle points, each representing a neuronal ensemble, forming a stable heteroclinic channel [26]. For the entries of the connectivity matrix, one chooses high inhibition from the previously active neuron to the currently active neuron and low inhibition from the currently active neuron to the next neuron that will become active:
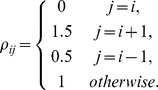
(Here 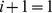 when 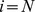 and 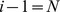 when 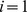).

**Table 1:** Variables used in the generative model.

Symbol	Meaning
*x* ^(*i*)^, *y* ^(*i*)^, *v* ^(*i*)^	Hidden states, *x* ^(*i*)^, *y* ^(*i*)^ and causal states, *v* ^(*i*)^
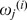	Normally distributed noise at the *i*th level
*κ*_1_, *κ*_2_	Rate constants: *κ*_1_ = 2, *κ*_2_ = 1
*λ*	Decay rate: 1/8
*ρ*	Connectivity matrix of the second level
*A*	Diagonal matrix with diagonal *a* = 0.2
*W*	Connectivity matrix of the first level
*I*	Direct input from the second level to the first level
*N*, *n*	Number of ensembles (*N* = 8) and (*n* = 6)

Note: This table lists the variables used in the generative, hierarchical model (see Equations 1 and 2 in Figure 1 and Model).

In the majority of the simulations below, we used 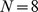 neuronal ensembles at the second level; longer sequences can be used as well, e.g., see the Recognition in a Noisy Environment simulation below and also [14]. Each second level ensemble, during its activation, sends a signal 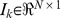 to the first level (see next section) designed to control the activation of the neuronal ensembles. The total signal sent to the first level by all second level ensembles at any time is a linear combination of the 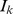
's: 
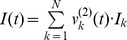
 where 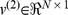 is the output vector in Eqn. 1. Note that, except during the transitions, only one entry of 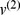 is close to one, all others are close to zero, which specifies the currently active population and therefore the dominating 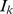. These 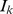
's are crucial for the model and the learning phase throughout the simulations above consists of reconstruction of these vectors.

#### First level: Spectro-temporal dynamics

We represent a collapsed form of lower level human auditory processing at the first level of our model. Each neuronal ensemble of the first level network represents spectral features of the cochleagram (see next section). The cochleagram consists of the firing rates of simulated auditory nerves, which are sensitive to specific frequency ranges. We encode these firing rates by the activity at the first level. When the neural network at the first level receives specific input 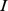 from the second level, the activity of the network is attracted to a global attractor encoding a specific spectral pattern in the cochleagram. As the input 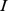 from the second level changes in a continuous, sequential fashion, this global attractor also changes continuously and neural activity of each ensemble encodes the cochleagram over time.

Here, we use a Hopfield network [32] to implement such dynamics. Hopfield network dynamics consist of stable equilibrium points that attract nearby orbits. Therefore, the itinerary of an arbitrary initial point evolves to one of these equilibrium points. Hopfield networks have been proposed to model associative memory, where each stable equilibrium point represents a memory item and an orbit attracted to such an equilibrium point represents a retrieved memory. In our model, at any given time, there is only one equilibrium point and this point changes depending on the sequential second level dynamics. We use the following equations for the first level of the generative model:
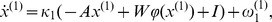


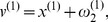
(2)where 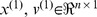 are hidden and causal states, respectively; 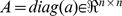 with scalar 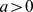, is a self-connectivity matrix, 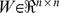 is an asymmetric synaptic connectivity matrix with entries 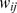 denoting the direction-specific connection strength from ensemble 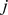 to 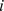, 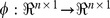 is a sigmoid function which we take as the *tanh* function applied component-wise, 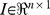 is the direct input from the second level, 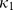 is a scalar and 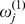 are normally distributed noise vectors. As previously described in [14], under mild assumptions on 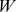 [33], one can choose the input vector 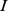 appropriately to create a global attractor with desired firing rate values. In the simulations, we show that it is also possible to learn the proper 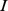 vectors from the speech stimulus using Bayesian techniques. Here, we use 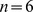 neuronal ensembles (see “Extensions and Limitations of the Model” in Discussion), which represent the reduced spectral output of the cochlear model. As a result, we obtain the necessary spectrotemporal dynamics where the sequential dynamics are provided by the second level and the mapping to the spectrum is encoded by the first level. A detailed explanation for the cochlear model is provided next.

#### Cochlear model: From sound wave to firing rates

The cochlea is a spiral-shaped peripheral organ of hearing in the inner ear which is a key component of the auditory system for translating acoustic waves into neural signals (see [34] for a review). Hearing starts with the travelling of sound waves through the ear canal and transmission of the resulting vibrations to the cochlea. The frequency specific representation of sounds comes partially from the differential stiffness of the basilar membrane, the elastic structure that extends through the cochlea. The base of the basilar membrane responds to higher frequencies and the other end, the apex, responds to lower frequencies.

Extensive research has been carried out to model the mechanism of the cochlea which is based on the fluid and the basilar membrane dynamics. Here, we use a classical model by R.F. Lyon [35] because it is simple and sufficient for our purposes, however note that more involved models exist in the literature (e.g. [36]–[40]). The output of the model, the cochleagram, is a time-frequency representation with values between zero and one which represent the firing rate of the corresponding auditory nerves (channels) at each time point.

The number of channels in the model depends on the sampling rate of the original signal and the frequency overlap between filters. For the results in this paper, we used the LyonPassiveEar function of the Auditory Toolbox [41] with default parameters that gives us 86 channels where these channels are ordered from higher to lower frequencies, i.e. the 1^st^ channel represents the highest frequency (∼8 kHz) and the 86^th^ channel represents the lowest frequency (∼0 kHz). Bayesian inference of 86 channels is computationally too expensive and therefore, we decrease the number of channels to six by averaging 14 channels at every time point (from channel 1 up to 84 = 14×6) and remove the last two channels which usually do not carry any significant signal. This gives us six neuronal ensembles' firing rate dynamics. As shown in Results, these six channels are sufficient to give good discrimination results between several speech stimuli. The time duration of these channels depends on the length of the stimulus and the decimation factor. Except stated otherwise, we scaled the duration of these six signals to 100 time units which allowed us to use the same number of second level ensembles for each stimulus. However note that in all figures, we used the original length of the corresponding stimuli in milliseconds along the *x*-axes for clarity.

### Learning and recognition

For a given speech stimulus *z* (preprocessed by the cochlear model) and a model *m*, the *model evidence* or *marginal likelihood of z* is defined by the conditional probability 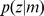 where the model *m* consists of all differential equations described before and priors for model parameters. The task for the module is to infer the corresponding causal states *v* and hidden states *x* at all levels as well as the parameters 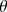, i.e. the 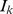
's that connect the levels, which we all together denote by 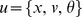. Therefore the goal is to estimate the *posterior density*, 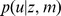, which describes the mean distribution of the variables as well as the uncertainty about them. We approximate the posterior in an indirect way:

The marginal likelihood of *z* is given by 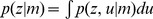 where 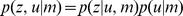 is defined in terms of the likelihood 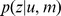 and the prior 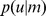. We approximate this intractable integral by introducing a *free-energy* term which is a lower bound for the marginal likelihood. It is straightforward to show that:
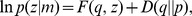
where 
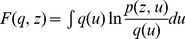
 is the free-energy, 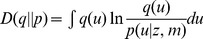 is the Kullback-Leibler divergence and 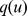 is the *recognition density*. Note that 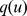 is an auxiliary function that we will use to approximate the posterior density. The divergence term *D* is nonnegative, 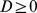, and 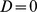 if and only if 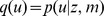. This means 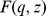 is a lower bound for 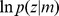, and if we can maximize 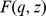, this will minimize 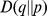 providing an approximation 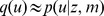 for the posterior density.

To find 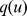 that maximizes 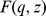, we make a Gaussian assumption about the form of 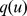, the so called Laplace approximation. Therefore we take 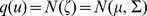 where 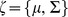 consists of the mode 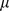 and the variance 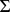. Now, the question turns into a maximization problem of the free energy with respect to 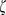:
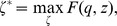
which gives the approximation for the posterior density 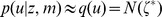. Note that the above maximization process is a simplified description and is only suitable for the time-independent *u* parameters (static case). When time-dependent states are involved, i.e. causal and hidden states, one needs to replace the free energy with *free action* 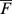 which is the anti-derivative of free energy in time, i.e. 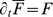. In this case, one aims at minimizing free action under the Laplace assumption. We note that time-dependent and independent variables can be handled concurrently and we refer the reader to [42] for details.

For all simulations in this paper, we used fixed prior variances for all states and parameters. The variances for the corresponding simulations are usually described in terms of the *precision*, 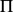, which is defined as the inverse of the variance, i.e. 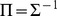. Therefore, a high prior precision for an internal state means that the dynamics are not allowed to deviate much from expectations provided by the generative model (top-down influence) whereas a low prior precision means the dynamics is relatively susceptible to (bottom-up) influences (wider standard deviation). Throughout the Results section, we report the log-precision values; the corresponding standard deviations can be computed by the formula: standard deviation = exp(−log precision/2).

The above maximization process can also be formulated in a hierarchical setting. Let us denote *all* hidden and causal states at level 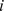 by 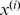 and 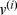, respectively. We also write 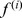 and 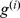 to describe the dynamics of the hidden and causal states at the 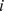 th level (see Eqns. 1 and 2): 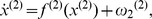


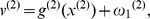


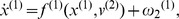


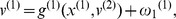
where 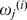 denotes the normally distributed fluctuations at the 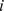 th level. Note that the second level causal states 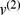 provide input to the first level while the hidden states 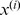 are intrinsic to each level. The preprocessed speech stimulus enters the system through the first level: 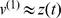. The optimization process described above, i.e. finding the optimum mode and variance for states and parameters, can be implemented in a message passing scheme [42] where the optimization problem turns into a gradient descent on precision-weighted prediction errors (see also Figure 2B):
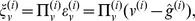


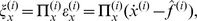
where 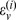 and 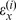 are causal and hidden prediction errors at the *i* th level, weighted by the causal and hidden precisions 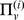 and 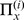 respectively; 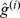 and 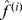 denote the internal predictions of the corresponding level for 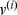 and 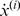, respectively. Internal predictions set the states to the right trajectory for future input. Therefore, it can be seen that as prediction error is minimized, internal predictions fit better to the external input. Intuitively, high precision for a variable means the prediction error is amplified and therefore only small errors are tolerated whereas low precision means large errors are tolerated and therefore the approximation to the states is *less precise*.

#### Neuronal network implementation

Finally, the Bayesian inference described above can be implemented in a neurobiologically plausible fashion using two types of neuronal ensembles. The modes of the expected causal and hidden states, 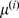, can be represented by the neural activity of *state* ensembles, while prediction error is encoded by the activity of *error* ensembles, with one matching error ensemble for each state ensemble. State and error ensembles interact within and between levels. The messages sent from second to first level state ensembles encode the expectations of the second level on the dynamics of the first level whereas error units at each level compare these expectations to the ongoing activity of state ensembles and compute prediction errors, which are passed on via forward and lateral connections. These error units can be identified with superficial pyramidal cells as they originate forward connections in the brain which correspond to the bottom-up error messages in our setting [43]. The sources of backward connections can be identified with deep pyramidal cells which encode top-down expectations of the state units. This message passing scheme efficiently minimizes prediction errors and optimizes predictions at all levels (for more details, see [43], [44]).

*Software note:* The routines (including commented Matlab source code) implementing this dynamic inference scheme, which were also used for the simulations in this paper, are available as academic freeware (Statistical Parametric Mapping package (SPM8) from http://www.fil.ion.ucl.ac.uk/spm/; Dynamic Expectation Maximization (DEM) Toolbox).

## Results

### A Bayesian model for learning and online recognition of human speech

In each module (see Model), learning and recognition of speech are simultaneous processes of adapting internal connections and inferring the speech message dynamics of the speaker. As in the brain, learning changes *parameters*, such as the synaptic connectivity, of the modules relatively slowly, whereas recognition is based on rapidly changing *states* of the system, such as the membrane potentials and firing rate [45], [46]. In all simulations below, there are two main tasks: (i) a learning task where the feedback parameters from second level to first are allowed to change and (ii) a recognition task where parameters are fixed and the model only reconstructs the hidden dynamics. In both cases, the model is given the appropriate precision settings from the beginning of the experiment and it either performs a learning task or a recognition task. A single learning step consists of learning one word by one module.

Both recognition and learning in a module starts with sensation; a speech sound wave (a single word for all but one simulations below), and after passing through the cochlea model this serves as a dynamic input to the module. The speech signal is preprocessed by the cochlear model and the dynamic output of the cochlear model, which we denote by a vector *z(t)*, reaches the first level of the module (Figure 1; for mathematical details see Model). Given this time-dependent vector *z(t)* and the two-level generative model (Equations 1 and 2 in Figure 1, see Model), each module infers the states of the first and second levels (recognition) and learns the connection weights from the second to the first level (*I*'s), see first line of Equation 2. To implement this, we used the Bayesian inference technique “Dynamic Expectation Maximization” [42].

Both levels of a module consist of neuronal populations that interact within and between levels. These populations encode expectations about the cochlea model dynamics, i.e. the sensory input, using the internal generative model described in the previous section. These expectations predict the neuronal activity (i.e., firing rates) at the next lower level, i.e., either at the cochleagram or the first level. The hierarchical inference uses top-down and bottom-up messages, which aim to minimize an error signal, the so-called prediction error. At any given time *t*, the input from the cochlear model, *z(t)*, is compared to the predictions at the first level which are produced by the generative model. During recognition, the prediction error is propagated to the second level where, again, prediction errors are computed using the generative model. Both levels adjust their internal predictions to minimize the prediction errors [42]. The module's expectation of how much an internal state will vary is a key parameter of the model: It is called “precision”. The precision determines how much error is tolerated at a specific level and we illustrate its relevance to speech learning and recognition in the next section.

During recognition, the second level forms predictions that are transmitted to the first level. This is only possible if the parameters for the backward connections between these two levels are appropriate; each module has to learn these parameters. In contrast to recognition, learning is not accomplished online because the information about parameters is obtained at a slower time scale, i.e., over the course of a complete stimulus (word) or repetitions of a stimulus. For learning, prediction errors are summed up for the whole stimulus duration and used after stimulus presentation to update the parameters. Therefore, as each module is exposed to repeated stimuli, the parameters are updated to minimize the prediction error accumulated over time, while states are updated in an online fashion to minimize temporally *local* prediction errors.

In summary, learning and recognition are realized as parts of the same inference scheme and work together to minimize overall prediction error. The necessary computations can be described as the dynamics of a hierarchically structured recurrent neural network operating online on the continuous speech input [43], [47]. For further details, see Model.

### Testing the human speech learning and recognition model

#### Learning speech

**Figure 3:**
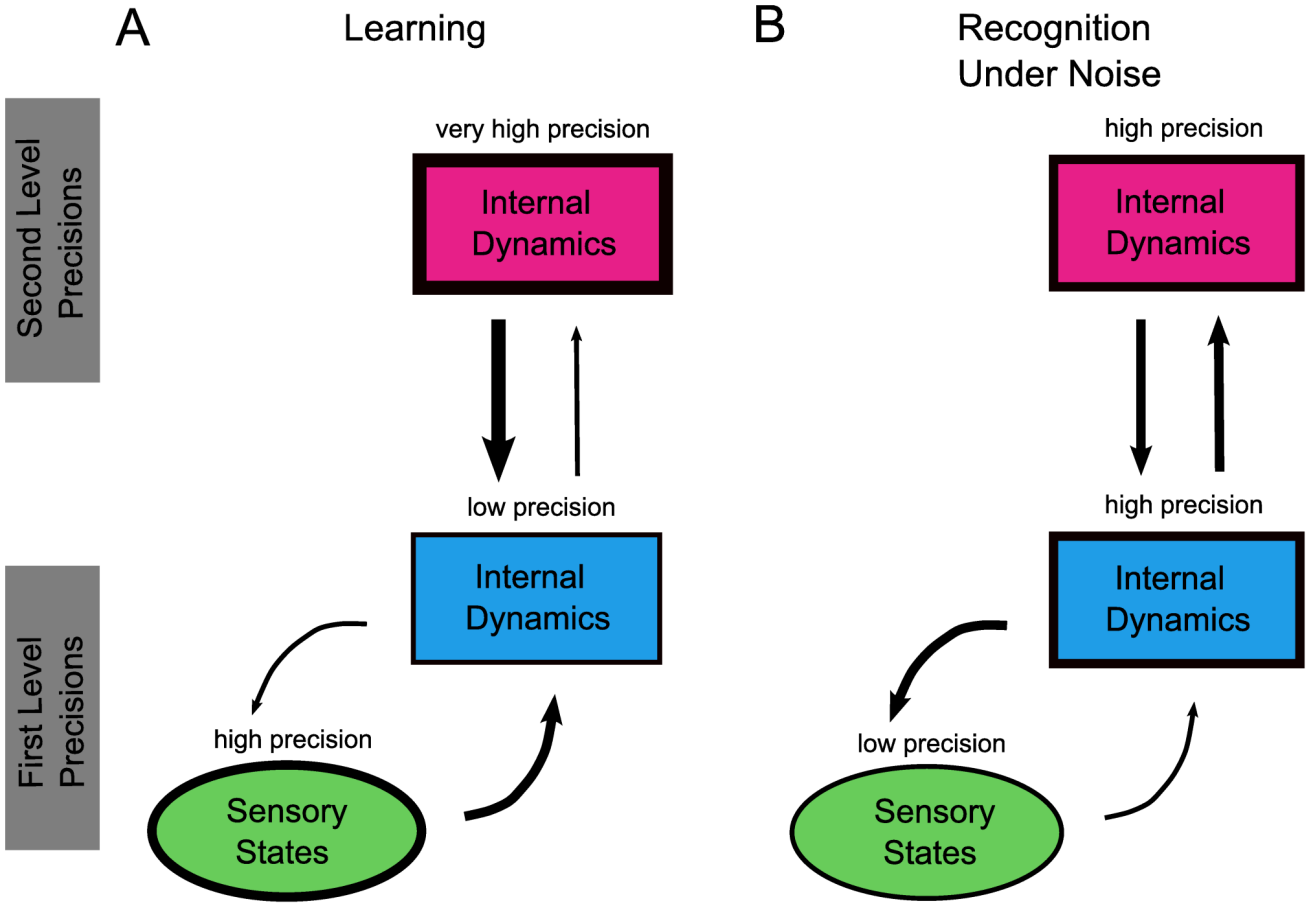
Schema of ideal precision settings, at the first and second levels of a module, for learning and recognition under noise. The precision of a population at each level is indicated by the line thickness around the symbols, and the influence of a population over another is indicated by arrow strength. **A**) During learning, the precision ratio at the first level (precision of the sensory states, i.e., causal states, over precision of the internal (hidden) dynamics) should be high. Consequently, the internal dynamics at the first level are dominated by the dynamics of the sensory input. At the second level, a very high precision makes sure that the module is forced to explain the sensory input as sequential dynamics by updating (learning) the connections between first and second levels (the *I*'s in the first line of Equation 2). **B**) Under noisy conditions, the sensory input is not reliable and recognition performance is best if the precision at the sensory level is low compared to the precision of the internal dynamics at both levels (low sensory/internal precision ratio). This allows the module to rely on its (previously learned) internal dynamics, but less-so on the noisy sensory input. For the exact values of the precision settings in each scenario, see Text S1.

**Figure 4:**
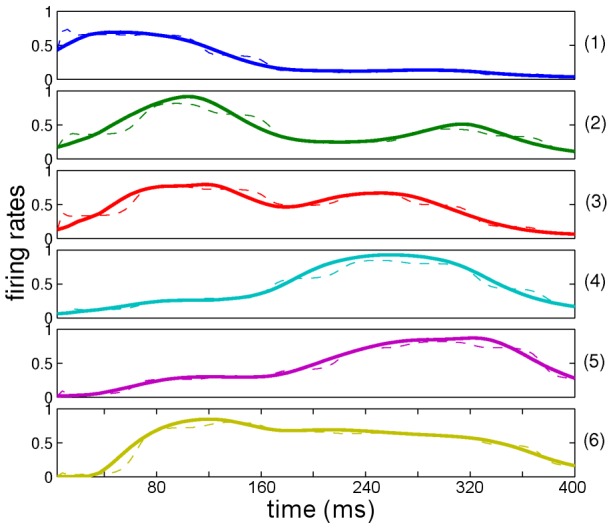
Generated neuronal network activity at the first level after learning. The solid lines represent the cochleagram dynamics obtained from the stimulus (the word “zero”, the same stimulus as shown in Figure 1) that the module had to learn. Neuronal activity was normalized to one. The dashed lines represent the neuronal activity generated by the module after learning and shows that the module has successfully learned the proper *I* vectors between two levels.

Before speech can be recognized, it has to be learned [48], [49]. We, therefore, first tested whether the model could learn to recognize words. For this, we used the sound waves of the words for digits zero to nine spoken by one speaker. We took the stimuli from a speech database (TI-46, www.ldc.upenn.edu), which is a standard benchmark test for speech recognition algorithms [50], [51]. We first put each module into learning mode, which is characterized by very high precision at the second level states and relatively lower precisions at the first level states (Figure 3A). This makes each module expect sequential dynamics at the second level and adapt states at the first level accordingly, using prediction error. In addition, at the first level, we set each module's precision for the sensory states, i.e., causal states at the first level (see Text S1) relatively high, while the internal dynamics at the first level have lower precision (Figure 3A). This precision ratio at the first level is crucial for learning: The relatively high precision forces each module to closely match the external stimulus, i.e., minimize the prediction error about the sensory input, and allow for more prediction error on the internal dynamics. To reduce these prediction errors, each module is forced to adapt the backward connections from the second level to the first level, which are free parameters in the model (the *I*'s in Equation 2). This automatic optimization process iterates until the prediction error can be no further reduced and is typically completed after five to six repetitions of a word. With this learning mode, we found that learning is typically completed after five to six repetitions of a word. In general, we found that precisions deviating from these settings will lead to either slower learning rates or no learning at all. To illustrate the quality of learning, we read out the internal model of each module by using the learned parameters to generate cochleagram dynamics and compared it with the actual stimulus that was learned. In Figure 4, we show a typical sample where the dynamics generated using the learned parameters (dashed lines) follow the cochleagram dynamics (solid lines) closely. Qualitatively, all words have been learned similarly well.

#### Word recognition task

After learning has concluded for each module separately and backward connections are fixed, we tested whether the agent showed high performance in a word classification task. We tested classification performance on a subset of the TI-46 speech database, which contained ten samples of ten words for digits (zero to nine) spoken by five female speakers, adding up to a total of 500 speech samples. To measure recognition performance, we used a cross-validation procedure, as is standard in speech recognition benchmark testing [51]: We randomly divided the 500 words into a training set (400 samples; 8 samples per digit and speaker) and a test set (100 samples; 2 samples per digit and speaker). In the training set, each module, one module for each digit sample, learned the backward connections between the second and first levels which gives us 400 parameter sets. To obtain ten speaker-independent and word-specific modules (one for each digit), we averaged these connections within digit. During the test phase, each of the 100 test samples, which had not been used during learning, were recognized by each of these ten modules while learning was turned off. For classification, we used a winner-take-all process (see Text S1) where the winner was the module with the *lowest* prediction error, i.e. the module which can best explain the sensory input using its internal model. The average Word Error Rate (WER; ratio of incorrectly classified test samples and the total number of test samples) was 1.6%. This is at roughly the same level as state-of-the art automatic speech recognition systems (Table 2).

**Table 2:** Word Error Rates (WER) for isolated digit recognition task reported in the literature for different recognition methods.

	DEM	LSTM	LSM	LSM 2	HMM	OT
WER	**1.6%**	2.0%	4.3%	0.2%	0.6%	2.4%

Note: DEM (Dynamic Expectation Maximization) is the recognition system used in this paper; LSTM (Long Short-Term Memory) network was introduced in [111], LSM (Liquid State Machine) with 1232 neurons was reported in [51] and was improved (LSM 2) in [18]. The results for the state-of-the-art speech recognition system using HMM (Hidden Markov Model) were reported in [18]. OT (Occurrence Time) features were used in [103].

Next, we tested whether the model is robust against noise. Following a noise reduction step at the cochlear level (see Text S1), the classification results in WER for different signal-to-noise ratios of 30 dB, 20 dB and 10 dB were 3.6%, 5% and 11.2%, respectively. The results compare well with the state-of-the art speech recognition system that has been tested on the same noisy input, i.e., using the liquid state machine (8.5%, 10.5% and 11.5% WER), respectively [51].

We next exposed the modules to situations that are quite typical for conditions under which humans perceive speech well but which pose severe challenges to automatic speech recognition schemes. These are variations in speech rate and accent, and cocktail party situations.

#### Variations in speech rate

The human auditory system shows remarkable flexibility for variations in speech rate [52], [53], whereas such variations pose a serious problem for automatic speech recognition models [50], [54], [55]. We, therefore, tested whether our recognition model is capable of dealing with time-compressed speech.

**Figure 5:**
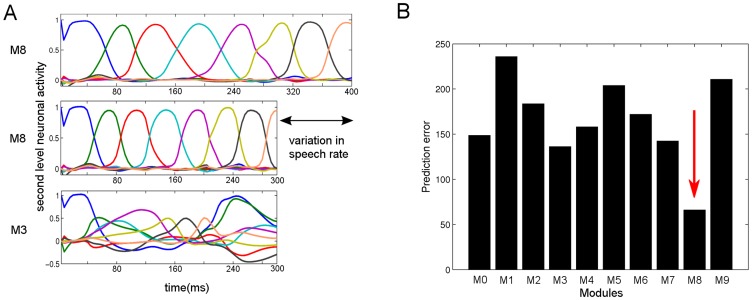
Invariance of the recognition model to variation in speech rate. **A**) The normal length stimulus “eight” (400 ms, top panel) has been learned and recognized successfully by the module “eight” (M8). For clarity, we only show the second level causal states (see Model). The same module (without any parameter adaptation) successfully recognizes a time-compressed version of the same stimulus (300 ms, middle panel). For comparison, the module trained on a digit “three” (M3) fails to reconstruct its expected dynamics when exposed to “eight” (bottom panel). **B**) The total prediction errors produced at the second level hidden states by ten different modules (M0 to M9), which were previously trained on the corresponding digits with normal length, are shown. All modules were exposed to the same 25% time compressed “eight” stimulus. Module M8 (red arrow) produces the lowest prediction error and shows that prediction error can be used for classification, even though the stimulus is time compressed.

We compressed the cochleagrams in time to induce variability in speech rate. We exposed a module, which was trained on a normal length of spoken digit “eight” (M8), to a sample compressed by 25%, without changing pitch. The results show that the module can recognize the time-compressed word (Figure 5A). Importantly, this recognition does not require any parameter learning. The module is inherently robust against time compression because it explains away the compression, using prediction error, by speeding up the sequential dynamics at the second level (see compressed dynamics at the second level in Figure 5A, middle panel). This works well because the module is informed, by its second level, about the sequence of dynamics expected for a specific word and temporal variation do not change this sequence. Importantly, even under compression, the recognition performance is still high. For example, a module that was originally trained on a normal-length “three” stimulus experiences a lot of prediction error when confronted with a compressed “eight” stimulus. This can be seen qualitatively by the confused sequential dynamics at the second level (Figure 5A, bottom). The module trained on normal length “eight” stimulus recognizes the correct sequence (Figure 5A, middle) and produces the lowest prediction errors for the time compressed “eight” stimulus, among all ten modules each trained on a different normal length digit (Figure 5B).

#### Recognition in a noisy environment

**Figure 6:**
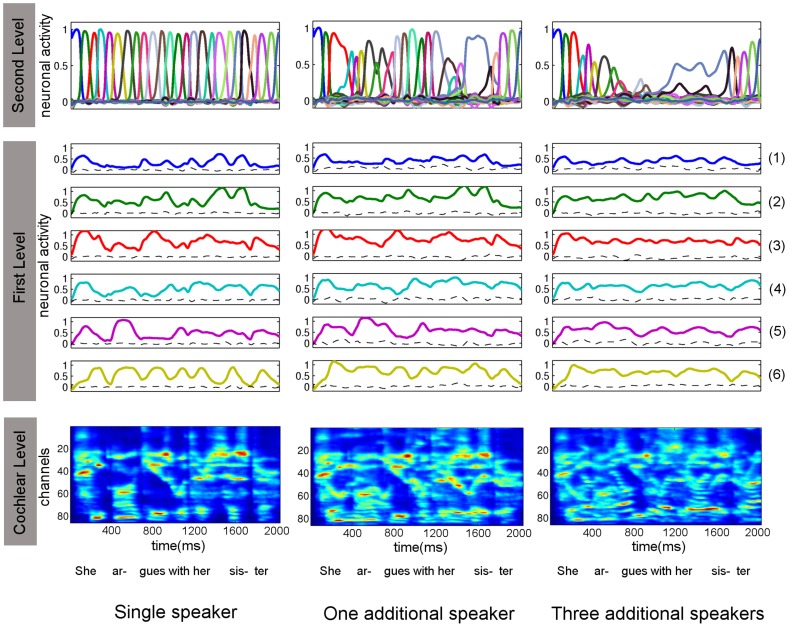
Performance of the recognition model in “cocktail party” situations. A module is trained on an auditory sentence (“She argues with her sister”) without competing speakers and tested for recognition of this sentence in three conditions: **Left column)** No competing speaker, **Middle column)** one competing speaker, and **Right column)** three competing speakers. Each column shows the second level dynamics, first level dynamics and cochleagram with arbitrary units in neuronal activation. Second level dynamics were successfully reconstructed for the single speaker and also, to an extent, for the speech sample with one competing speaker. In the case of three competing speakers, the module was not able to reconstruct the second level dynamics completely, but showed some signs of recovery at the beginning and at the end of the sentence. Note that the increasing difficulty in reconstruction of the speech message from one to three speakers is not reflected in the prediction errors at the first level (dashed lines), but becomes obvious at the second level.

Humans are able to concentrate on a specific speaker's voice when there are other competing speakers, as typically experienced at a cocktail party [56]–[59]. This is often tested with sentence-long stimuli with an increasing number of speakers. Here we used the target sentence “She argues with her sister.” (stimulus taken from [58]) and presented it to a module without background speaker, with one background speaker, and with three background speakers. Therefore, a module represents the dynamics of a whole sentence instead of a single word as in the previous simulations. The background speakers speak different sentences, and have a loudness level that corresponds to a location in space that is twice as far away from the listener as the target speaker. The module, as expected, is able to reconstruct the second level dynamics perfectly when it is exposed to the clear stimulus without background speakers (Figure 6, left column). It also reconstructs the target sentence dynamics when there is one additional speaker in the background (Figure 6, middle column). The second level always shows the correct order of activation even though some of the elements of the sequence are slightly misplaced in time when the background speaker masks the target (Figure 6). This is immediately corrected once the target sentence is again discernible in the cochleagram, i.e., when interference with the target sentence becomes small enough. In humans, such periods of recognition may be useful to help recognize the target sentence [58]. The module can very roughly reconstruct the second level dynamics and the correct order of activations when there are three background speakers (Figure 6, right column); the dynamics can be recovered at the beginning and towards the end of the sentence. These simulations suggest that the module uses expectations about sequential dynamics, i.e., dynamic predictions, at the second level to recover a target sentence from corrupted sensory dynamics.

### Adaptation and learning of speech

In the following two sections, we describe how we tested the hypothesis that the prior precision setting of a module is fundamental for understanding the learning of speech. This hypothesis follows from the construction of the module where only two different interpretations of suboptimal speech recognition exist: (i) the sensed speech is noisy, or (ii) the module's internal model is not appropriate and needs to be adapted. This is why the precision ratio at the first level, i.e., a module's expectation about how noisy speech dynamics are relative to its internal dynamics, is fundamental for learning. A precision setting as shown in Figure 3A will effectively exclude the module's assumption that speech is noisy; rather it will rely on the assumption that speech is sequential based on a high precision of the dynamics at the second level. This will prompt the module to adapt its internal speech model.

#### Accent adaptation

Foreign accents are often a cause of severe variations in spoken language. Behaviorally, recognition of foreign-accented speech can affect the comprehension of words [60] and increase the processing time of listeners who are used to unaccented speech [61]. However, relatively brief exposure (between 2 and 64 sentences) to foreign-accented speech improves listeners' recognition accuracy [62] and efficiency, measured in terms of error rates and reaction times [63].

Here, we show how this rapid accent adaptation can be implemented by the present model and how behavioral differences in adaptation can be explained. By “adaptation” we mean that the learning of the parameters in a module proceeds from a previously learned parameter set (base accent) as opposed to learning from scratch in the “Learning speech” simulation. Therefore, adaptation can be understood as slight changes of the backward connections instead of learning a completely new word.

**Figure 7:**
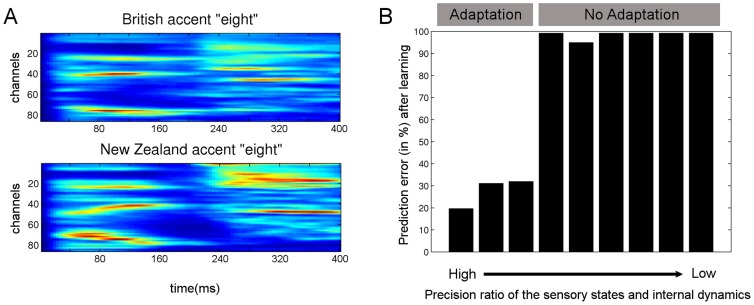
Accent adaptation of the recognition model. **A**) The cochleagrams represent two utterances of “eight”. A module originally learned the word “eight” spoken with a British (North England) accent (top) and then recognized an “eight” spoken with a New Zealand accent (bottom). **B**) The module trained on the British accent was allowed to adapt to the New Zealand accent with the corresponding precision values for the first level sensory (causal) and internal (hidden) states (sensory log-precision: 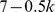 and internal log-precision: 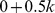 where 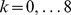 from left to right). For each precision ratio, we plotted the reduction in prediction error (of the causal states, see Model) after five repetitions of the word “eight” spoken with a New Zealand accent. As expected, accent adaptation was accomplished only with high sensory/internal precision ratios (resulting in greatly reduced prediction errors) whereas no adaptation occurred (prediction errors remained high) when this ratio was low.

We trained a module to recognize the speech stimulus “eight” spoken with a North England accent (Figure 7A, top) and tested recognition for an “eight” spoken by a different speaker with a New Zealand accent (Figure 7A, bottom; stimuli taken from www.soundcomparisons.com). On first presentation of the word, the module experiences increased prediction error during recognition of the accented word, i.e., it would perform worse in a word recognition test. We hypothesized that a crucial criterion for whether a module can, or cannot, adapt to an accent, is its prior precision of the sensory states, i.e., how noisy the module expects the sensory input to be. If this precision is low compared to the precision of the internal dynamics (“recognition mode”, as shown in Figure 3B), no adaptation is induced, because the module accepts the slight variations due to the accent as noise on its sensory input. If, however, the module expects input to be sensed with high precision, an accented word causes the module to adapt its internal model, i.e., its backward connections from the second to the first level. This is, from the module's view, the only way to explain the unexpected variations in the input (“learning mode”, as shown in Figure 3A). We tested this explicitly by controlling the ratio of the module's prior precision of the sensory states and internal dynamics (sensory/internal precision ratio) at the first level of the model. As expected, we found that only a module that has a high precision ratio at the first level (learning mode, Figure 3A) rapidly adapts to accented speech (Figure 7B). With the three highest precision settings, this was achieved after only two to three iterations. For lower precision ratios, practically no adaptation occurred. This suggests a potential mechanism for the inter-individual variability to accent adaptation: agents, and potentially also humans, attending to sensory detail can adapt to accents while agents/humans who literally explain deviations as background noise cannot adapt to accents. However, it should be noted that this is a rather simplistic explanation which has emerged as a consequence of our simulations and does not explain all aspects of accent adaptation. It should be considered as an interpretation of the optimum precision settings obtained through simulations.

#### Speech learning: Qualitative modeling

Can the precision setting also explain a re-learning of speech, as for example in second language learning? People start learning second languages at different ages. The age of second language acquisition is an important factor for being fluent in the new language [64], [65]. This age factor is behaviorally relevant when second language learners are asked to recognize words or sentences embedded in background noise [66], [67]. Here, we tested whether the present model could be used to qualitatively model behavioral results [67]. If this were possible, it would imply that the model represents a potential computational mechanism for explaining the importance of age in language learning.

**Figure 8:**
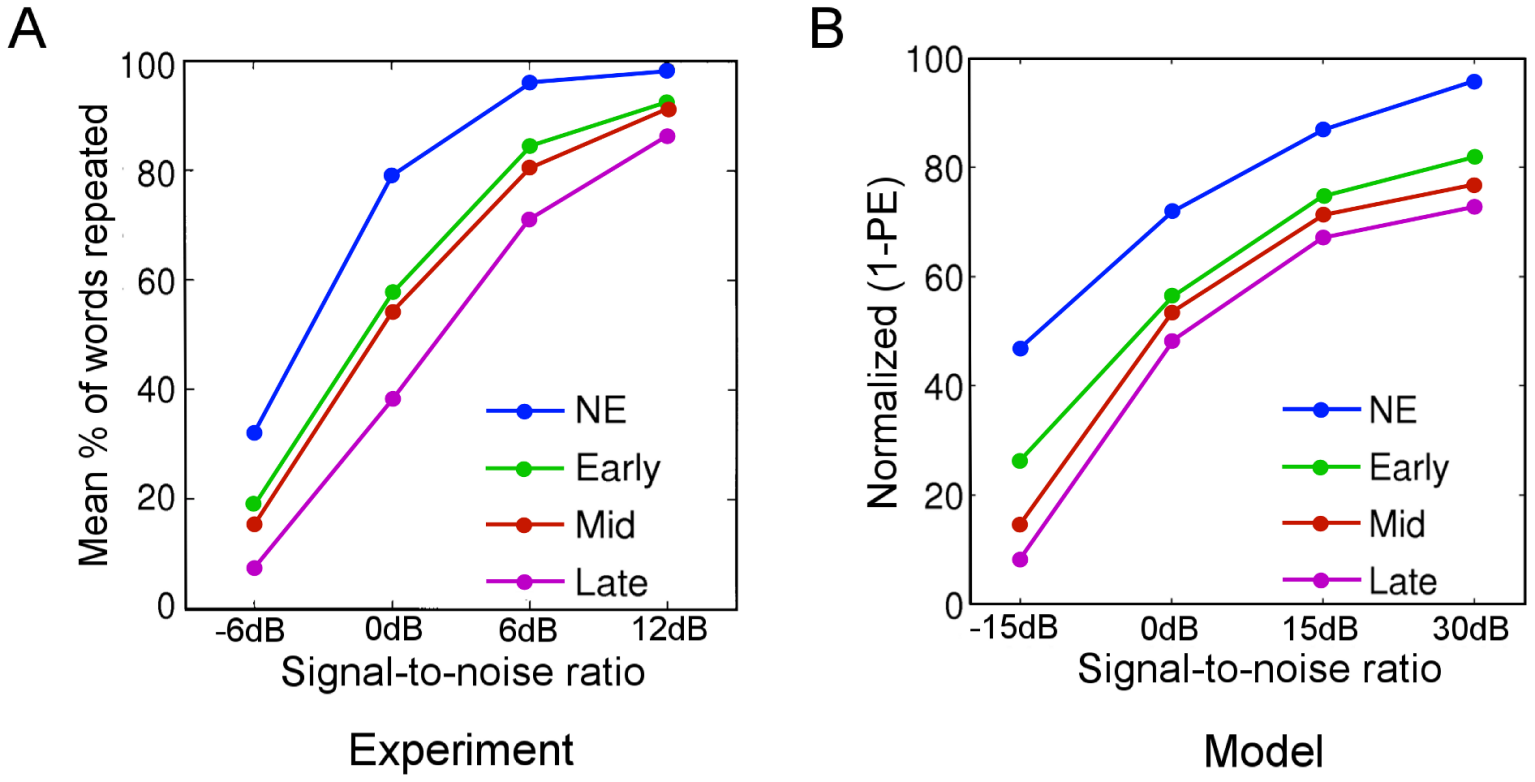
Qualitative modeling of experimental results in second language learning. **A**) The behavioral results of an experiment [67] for the recognition of English words by three groups of native speakers of Italian who differed in their age of arrival in Canada: Early, Mid and Late arrival groups, also compared to a native English speaker (NE) group. Participants were asked to repeat as many words as possible after they heard an English sentence. Sentences were presented at different signal-to-noise ratios given in decibels (dB). Adapted from [67]. **B**) The results of the learning and recognition simulations where we used the same speech samples as in the Word Recognition Task. The different age of arrival was modeled with different precision ratios at the first level. Recognition accuracy is measured in terms of normalized, total causal prediction errors during recognition relative to a baseline condition of −30 dB noise, i.e., recognition accuracy = 100*[(baseline prediction error-test prediction error)/baseline prediction error]. Note that we used different signal-to-noise ratios than the original experiment because best recognition results with our model were obtained at 30 dB, which corresponds to almost ideal recognition results in humans around 12 dB, and we scaled the remaining ratios accordingly. Each symbol represents the average recognition accuracy obtained from 10 digits where the stimulus was masked with noise at given signal-to-noise ratios.

A previous study examined the recognition of English words by three groups of native Italian speakers with different mean age of arrival (mAOA) when immigrating to Canada [67]: An early group (mAOA of 7 years), a mid group (mAOA of 14 years) and a late group (mAOA of 19 years). In addition, there was a control group of native English speakers. The stimuli consisted of ten English sentences presented at four different signal-to-noise ratios (−6, 0, 6 and 12 dB). The participants repeated as many words as possible after each presentation of a sentence. Significantly higher recognition accuracies were obtained for early, as compared to the mid and late groups, and the native group performed significantly better than all immigrant groups (Figure 8A).

We simulated second language learning using the present model to explain these behavioral results. As second language, we used digit words and simulated different ages of arrival by using different precision settings (from high first level sensory/internal precision ratio for native English speakers to progressively lower ratios for early, mid and late groups, see details in Text S1). The recognition results compared well with the experimental results (Figure 8B). The recognition accuracy improved with increasing signal-to-noise ratios in all groups and the native speakers recognized more accurately at all noise levels followed by the early, mid and late groups. These results suggest the computational mechanism for the behavioral results found in the four groups: The longer someone is exposed to his/her native language, the more precise the expectations could be about the brain's internal dynamics when recognizing speech. This high precision would be counter-productive when learning a second language because internal dynamics are not learned optimally: the agent, i.e., the brain, would rather explain away prediction error by assuming that speech of the second language is relatively noisy as compared to speech of the first language. However, it should be noted that there is no experimental evidence for such a claim yet, i.e. that the words in a second language are considered to be noisy in late learners, and this point should be taken as an interpretation of our computational results. In fact, as pointed out by one of the reviewers, many studies have concluded that the amount and variability of second language input [68]–[70] as well as the frequency of using the native language during learning [71] have considerable influence in the age of acquisition effects.

## Discussion

We have developed a novel model of speech learning and recognition that is implemented as a hierarchically structured recurrent neural network. The core structure of the network was taken from a birdsong model that was based on key experimental findings in songbirds [14]. We found that the resulting computational model achieves very high recognition performance when recognizing words directly from speech sound waves, both under ideal noise-free and noisy conditions. In addition, the model deals well with situations in which automatic speech recognition usually fails, but humans still perform well: adaptation to varying speech rate and competition by multiple speakers. The model is also able to explain inter-individual differences in accent adaptation, as well as age of acquisition effects in second language learning.

### Sequential dynamics in song and speech recognition

In songbird studies, temporally precise sequential activation of neurons in a high level structure, HVC, has been observed during singing [22], [23] and the same area has also been shown to be involved during recognition of songs with similar precise activations [6]. It has been suggested that Broca's area in the inferior frontal gyrus (pars opercularis) in humans corresponds functionally to HVC in songbirds [2], [72]. Similar to HVC, this area in the human inferior frontal gyrus is involved in recognition and production of speech. It has been implied in sequence perception and in providing top-down predictions to auditory speech processing areas (for a review see [73]). We suggest that this is a candidate area for including precise sequential activation of neurons, as modeled by dynamic sequences at the second level of the present model. The existence of sequentially activated, temporally precise, neuronal ensembles in the cortex has been proposed previously [74] and provides an explanation for findings of a precise spike timing which have been observed in experiments in different species, e.g. [75]–[77]. There do not seem to be equivalent neuronal studies in humans; however, speech processing activity, as observed with magnetoencephalography, has been explained as large-scale sequential activity [78]. Based on the results of the current paper, we predict that such sequential activations in the human brain, expressed at a microscopic level, e.g., in spike timing, are crucial in organizing the auditory information coming from the lower areas to form the dynamic percept of phonemes, syllables and words.

Even though the second level ensembles in the proposed model are encoded as temporally regularly spaced sequences in the generative model, we showed that during recognition (see Variations in Speech Rate simulation) they have the flexibility to activate earlier or later according to the spectrotemporal features they are tuned to. This fits well with a recent study [79] where the authors presented evidence that HVC activity is timed to particular time points of motor gestures during song production. The current generative model does not include a vocal tract mechanism [80]; however such a mechanism could be readily incorporated with an extra level at the bottom of the hierarchy (see [14] for an example).

To model neurobiological findings in songbirds, we used an advanced Bayesian inference scheme using recurrent neuronal networks. To our knowledge, this type of model has not been used before, neither in human speech recognition nor automatic speech recognition. One advantage of this approach is that recognition is performed in a brain-like fashion on continuous sensory dynamics, in contrast to a standard hidden Markov model operating on discretized input [16]. In addition, the present model can be used, as we have demonstrated, to incorporate experimental birdsong findings by specifying a hierarchically structured, generative model based on nonlinear dynamical systems and translate the resulting model to human speech.

### Comparison to other speech processing models in neuroscience

Our approach is unique in the sense that we use a hierarchy of nonlinear dynamical systems as a generative model to provide an online Bayesian inversion mechanism of human speech. Many other computational speech and word recognition models have been proposed that are both neurobiologically plausible and can explain experimental results [19], [20], [81]–[87]. These models typically focus on the word selection process rather than on how relevant spectrotemporal features are extracted from the sound wave. For example, most of these models assume that relevant phonemic features have already been extracted from the sound wave and arrive in regular intervals. This is in distinction to the present approach which models the extraction of relevant speech features from a noisy, continuous sound wave with varying speech rate. An example for these word selection models is the hierarchical TRACE model [19], [88]. There are three key differences between the TRACE and the present model. First, there is no learning in TRACE: the model parameters have to be manually set to enable recognition. Second, TRACE does not represent precision, which, as illustrated above, may be important to explain phenomena in both perception and learning. Third, TRACE is based on the competition of relatively simple processing units, and, therefore, is unable to identify local mistakes or mispronunciations; it returns the most probable word. In contrast, the present model can monitor such mismatches in an online fashion using the prediction error. This enables the processing of slight differences in pronunciations, as, for example, when the proposed model was used to adapt to speech with an unusual accent.

Another widely known model is the Shortlist model and its Bayesian version Shortlist B [20], [21]. Both models have most of the functionalities of the TRACE where information is processed in a feed-forward fashion. The Bayesian approach introduced in Shortlist B [21] illustrates a useful way to combine prior information such as word frequency with the likelihood function of the speech input. This demonstrates the interplay between the priors and the precision of the agent (called reliability in [21]). This is similar to the present model, where a differential setting of the precision parameters causes either recognition or learning mode of the sensory input (see Figure 3). The main differences between the present model and Shortlist B are that (i) Shortlist B does not allow for speech learning, (ii) Shortlist B assumes that phonemic features have already been extracted by some preprocessing stage while we explicitly model this stage using the cochleagram, and (iii) Shortlist B has been formulated as a feed-forward model only while the present model explicitly uses top-down influence to improve recognition of noisy input.

A different category of models has focused, like the present model, on the processing of auditory stimuli by single neurons or network of neurons [18], . For recognition, these models typically have to wait until the end of the stimulus to obtain all required neuronal responses. This is different from human performance where recognition can be performed online while the stimulus is received. This online recognition using predictions is also a hallmark of the recognition model proposed here, where the accumulated prediction error can be used for recognition anytime during stimulus presentation.

Recently, so called reservoir computing techniques using recurrent neural networks have been used for speech recognition [18], [51], [89]–[92] and provide excellent recognition results. Typically, these results are achieved with large networks of hundreds of neurons. This is different from the present study where we used few neurons for word recognition, i.e. just eight neurons at the second level and six neurons at the first, for each module. It would be worthwhile to consider recurrent networks as used in reservoir computing as a generative model in a Bayesian approach to better understand the mechanism underlying high recognition performances in reservoir computing.

### Precision: Link to neurotransmitter

Using simulations, we have shown that the precisions of the states (i.e., how certain the agent is about its internal states and dynamics) at different levels of the hierarchy are fundamental to learning and recognition of speech. Here, we fixed the prior precisions at each level to use appropriate precision settings during learning and recognition. The actual mechanisms in the brain for achieving such context-dependent optimum precision values are not known. Neurobiologically, cholinergic neurons (whose main neurotransmitter is acetylcholine, ACh) are known to be involved in the modulation of perceptual processes [93], [94]. It has been proposed that ACh may have the role of reporting on uncertainties of internal estimates and that high levels of ACh should correspond to faster learning about the environment and enhancement of bottom-up processing [95]. Such claims fit well with the present study since we found that increased precision about sensory states is ideal for learning speech as it enhances the influence of sensory information; whereas, learning deteriorates with decreasing precision ratios (Figure 3 and 7). We predict that increased levels of ACh may enhance the learning of novel auditory stimuli by suppressing top-down effects caused by a relatively low precision of internal dynamics; however, this should, in parallel, also disrupt perception of noisy stimuli since top-down information is crucial in cocktail-party like situations, (right column of Figure 6). Such claims could be tested with a behavioral study while manipulating the neurotransmitter levels pharmacologically [96].

### A novel analysis tool for neuroimaging experiments

The proposed model makes a computational link between sensory input (i.e., the speech sound wave) received by subjects and the dynamics of their hypothesized internal representation [97]. In particular, we found that the prediction error is a key quantity that can be used to achieve high performance in speech recognition. This quantity can be used in novel computational analysis techniques for speech recognition neuroimaging experiments: The idea is to use the dynamics of the module's internal prediction error when receiving speech input as a predictor for neuronal activity in human subjects receiving exactly the same stimuli (see [98] for a similar study). This modeling approach would enable one to identify the exact computational role of specific areas in the well-established speech recognition system. In addition, this approach can be applied to speech learning studies (accent adaptation and second language learning), where one would use the module's prediction error experienced during learning to predict subject's changing brain activity during learning and estimate the precision parameters which subjects use. This may be done using either a voxel-wise regressor-based approach, or a network analysis (Dynamic Causal Modeling [99],[100]). For example, one may estimate the changes in effective connection strength in a network including the inferior frontal gyrus and primary auditory areas during accent adaptation or for speech recognition under different levels of noise. It would also be revealing to include a variety of precision settings as an experimental condition in studies that specifically test the hierarchical predictive coding hypothesis in the auditory cortex [101].

### Extensions and limitations of the model

Here, we only used six neuronal ensembles to represent the cochleagram in six frequency channels. This resolution is comparable to the low number of spectral channels used in cochlear implants [102]. Nevertheless, the model provided competitive recognition results (Table 2). We found that this performance drops if only four channels are used, but we did not explore this using more channels because the required computational power quickly increases with the current implementation (with complexity 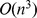). This computational issue could be resolved by parallel ensemble-specific computations, which would be another step towards biological reality and probably improving recognition rates further. It would also be worthwhile extending the cochlear features in the present model with other biologically plausible preprocessing steps, such as occurrence times, which encode the onsets and offsets of specific features [50], [103].

It is important to notice that the current model is not entirely specific to speech but can also be used to recognize other sound sequences such as music. In a future project, we will therefore make the model more speech-specific and extend the current model by including a vocal tract model in addition to the cochlear processing. This would make the inference more sensitive to relevant features in human speech and thereby improve recognition. Moreover, such a vocal tract mechanism would be beneficial for recognizing speech from different speakers since speaker-specific parameters can be included in the vocal tract model and constrain the recognition dynamics. This would allow the model to identify the similarities between words even if they are spoken by differently sounding speakers and therefore have little acoustic overlap. Such a model can also be used to qualitatively model specific findings at a phonemic level [69].

It is also worth mentioning that we assumed a fixed second-level connectivity matrix in the model (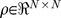 in Eqn. 1) which produces expectations about sequential dynamics by winnerless competition. We assumed here that such a structure already existed at the higher levels. It may also be possible to learn these specific connections from scratch; however, we expect that one would need relatively informative priors about these parameters to limit the search space.

Moreover, the generative model could be extended by adding extra levels to the hierarchy of nonlinear dynamical systems. This would allow the modeling of sequences of phonemes and syllables [104], or even sentences as sequence of words [105]. This can be done either using the technique proposed in the present paper or by using carefully designed nonlinear dynamical systems, as exemplified in [106]. Such detailed sentence level representations could be used to model syntactic experiments as shown in [107]. Using hierarchies, it would be useful to model the competition between possible alternative descriptions that emerge from partial stimuli where predictions provide constraints for the appropriate dynamics and therefore stable perception [108]. Such a hierarchical extension would be ideal to model the word selection process as exemplified in Shortlist B [21] while using real speech (sound waves) as input. Finally, the proposed learning and recognition technique could be extended to also estimate dynamically the precision values based on techniques as employed by [109]. This would allow the model to fine-tune the precision settings as a part of the optimization process. Currently, one still needs to provide the prior precision settings to inform the model about the context of the experiment, i.e. whether it is a learning task or recognition task.

### Conclusion

We proposed a computational model using a hierarchy of nonlinear dynamical systems and Bayesian online filtering for learning and recognizing sound sequences such as speech. This model was derived from a neuronal model for recognition of birdsong. It achieves high speech recognition performance and explains several auditory recognition phenomena, as well as behavioral data. This work has three implications. First, it shows that human speech and birdsong recognition systems may share similar computational components. Secondly, the competitive performance, even under adverse conditions, suggests that it may be used to optimize automatic speech recognition. Thirdly, the neurobiological plausibility of the model enables the generation of predictions for neurobiological, e.g., neuroimaging, experiments.

## Supporting Information

Text S1 Here we describe further details about the simulations presented in the Results section.
(PDF)
