# Supplementary material for: From Birdsong to Human Speech Recognition: Bayesian Inference on a Hierarchy of Nonlinear Dynamical Systems
Source: PLoS Comput Biol. 2013 Sep 12;9(9):e1003219. doi: 10.1371/journal.pcbi.1003219 (PMC3772045; doi:10.1371/journal.pcbi.1003219)
Supplement: Text S1 — Here we describe further details about the simulations presented in the Results section. (PDF) [file pcbi.1003219.s001.pdf]

Supplementary Text S1

Modeling and implementation details

In this section, we provide further details of the simulations described in Results.

Learning speech

For single word experiments, we normalized the duration of each cochleagram to 100 time units (~half a second) and we used eight neuronal populations at the second level to represent a word. Note that this normalization is not necessary at all for learning or recognition; rather we used it to meaningfully compare the recognition performances as described in the Word Recognition Task.

For minimal assumptions about what values the I_k vectors should take, we use shrinkage (zero mean) priors for I_k's and adapt them by learning using Dynamic Expectation Maximization described in the Model section.

To learn these connections, we used a very high precision for the states at the second level (log-precisions of 16 and 24 for causal and hidden states, respectively), and used a relatively lower precision at the first level (log-precisions of 7 and 0 for causal (sensory) and hidden states, respectively).

Word Recognition Task

For recognition, we used high precisions for the hidden states (log-precisions of 12 and 20 for the first and second levels, respectively) and relatively low precisions for the causal states (log-precision of 3 for both the first and second levels) because modules have already learned the appropriate internal dynamics. We used the following process to make a decision about which word was presented: For a specific test sample, each of the ten modules which have each learned a specific digit from zero to nine produced prediction errors for hidden and causal states at the two levels when recognizing input. For each module, we accumulated the prediction error of the causal states over time obtaining a total prediction error. We excluded the prediction error from the hidden states because these were small (due to the high prior precisions), as compared to the causal states. We used a winner-take-all process where the winner was the module with the *lowest* prediction error, i.e. the module which can best explain the sensory input using its internal model. This process (accumulation and min-operation) can be implemented by a higher level area and we call this structure which consists

of several modules, the agent. All reported error rates are the average rates obtained from a 5-fold cross validation where we used different test sets each time for accurate word error rate (WER) results.

After recognition of clean speech, we also tested the agent's performance for noisy speech recognition. Following the learning procedure with clean speech samples, i.e. as measured under ideal recording conditions, we added white noise to each of the 500 sound wave samples to increase the difficulty of the recognition task. Even though white noise is stochastic and is added to each speech sample independently, an average pattern caused by white noise can be easily detected and learned from the resulting cochleagrams. We modeled this as a first feed forward step where a simple subtraction removes this average pattern of white noise before the preprocessed sensory input is passed forward to the recognition module. In particular, white noise added a constant to each frequency band with decreasing amplitude the higher the frequency band. Such noise reduction processes are also observed in humans through active cochlear mechanisms such as the electromechanical feedback of outer hair cells [1,2]. Without this preprocessing step, the WER for noisy stimuli are: 7.4% at 30 dB, 26.8% at 20 dB, and 56% at 10dB which is comparable to [3].

Variations in speech rate

We exposed the recognition model, which was trained on a normal length spoken digit (400 ms), to a sample compressed by 25% (300 ms). We used relatively low precision for the states of the second level (log precisions of 5 and 0 for the causal and hidden states, respectively) since the module must be able to follow the unexpected fast stimulus.

Recognition in a Noisy Environment

Since 'Cocktail Party' stimuli are longer than the single words used above, we used 25 neuronal ensembles at the second level. Moreover, we used slightly higher precisions for the causal states during the recognition of the clear sentence and decreased this precision progressively for noisier stimuli as the stimulus becomes less reliable.

Second language learning

In the experiment, the recognition accuracy was computed as the percentage of correctly repeated words. We used the digit samples of the Word Recognition Task as stimuli. Similarly, ten modules, one for each digit, were trained on clear speech samples. The test

stimuli were noise-masked versions of the clear stimuli (pink noise as used in the experiment) at signal-to-noise ratios: -15, 0 15 and 30 dB. We picked these ratios since best recognition results with our model were obtained at 30 dB which corresponds to almost ideal recognition results in humans around 12 dB and we scaled the remaining ratios accordingly. To test the hypothesis that the internal precisions of a module explain how well a second language is learned, we modeled the different AOA groups by learning with different precision settings for the sensory states (C1) and hidden states (H1) at the first level. To model the effect of increasing AOA we used a decreasing C1/H1 ratio which should result in less efficient learning according to the results in the Accent Adaptation simulation. Specifically, we used, the following log-precisions: Native speakers (C1 = 6.75, H1 = 0.25), Early group (C1 = 4.75, H1 = 2.25), Mid group (C1 = 4.5, H1 = 2.5) and Late group (C1 = 4.25, H1 = 2.75). After learning, we exposed each module to its corresponding digit stimulus at different signal-to-noise ratios and computed the accumulated causal prediction errors. To model behavioral measurements, we used the normalized 1 – prediction error, i.e. 100*[(baseline-prediction error)/baseline], where baseline stands for the baseline prediction error which is obtained from the recognition of stimuli with high noise, i.e. -30dB.

References

1. Kim S, Frisina RD, Frisina DR (2006) Effects of age on speech understanding in normal hearing listeners: Relationship between the auditory efferent system and speech intelligibility in noise. Speech Communication 48: 855-862.
2. Liberman MC, Guinan JJ (1998) Feedback control of the auditory periphery: Anti-masking effects of middle ear muscles vs. olivocochlear efferents. Journal of Communication Disorders 31: 471-483.
3. Zavaglia M, Canolty RT, Schofield TM, Leff AP, Ursino M, et al. (2012) A dynamical pattern recognition model of gamma activity in auditory cortex. Neural Networks 28: 1-14.
